# Handle with care: optimising organ transplantation

**DOI:** 10.1016/j.ebiom.2020.103135

**Published:** 2020-11-16

The 2020 Nobel Prize in Physiology or Medicine was awarded to Harvey Alter, Michael Houghton, and Charles Rice for the discovery of the Hepatitis C Virus (HCV), the causative agent of hepatitis C. WHO estimates that, worldwide, 71 million people are currently living with chronic HCV infections which, if left untreated, can lead to cirrhosis and hepatocellular carcinoma, both of which can cause liver failure. At this advanced stage, the best treatment is liver transplantation.

Globally, the number of patients in need of new organs vastly outnumbers successful transplantation operations. As of September, 2020, the US Health Resources and Services Administration stated that 109 000 US patients needed an organ donation (with livers being the second most required organ after kidneys). However, only 40 000 successful transplants were done in the USA in 2019. A similar story is seen worldwide.

One way to address this concern is to increase the number of potential donors. In May, 2020, England altered its rules on organ procurement; from this date, all adults in the country are considered to have agreed to become donors upon death unless they opt out. Similar policies are present in other countries such as Israel, Chile, and Spain (who have had the policy since 1979). Increasing the number of potential donors is unlikely to solve the problem entirely, as not all donated organs are suitable for transplant and not all transplantation procedures are successful. A quartet of manuscripts published in the recent October Issue of *EBioMedicine* highlight research aimed at improving transplantation efficiency.

Organs accrue damage throughout an individual's life, potentially preventing transplantation. One of the most important metrics of liver health is the proportion of hepatocytes harbouring large fat vacuoles, known as steatosis. Fattier livers are more likely to undergo dysfunction if transplanted. Manuela Cesaretti (Beaujon Hospital, Clichy, France), writing in *Liver Transplantation* in 2019, stressed the need for reliable tools to measure steatosis. Work led by Lulu Sun (Washington University School of Medicine, MO, USA) in our October issue reported on a deep learning algorithm (DLA) that calculates steatosis from images of frozen liver biopsies. The DLA estimates were more accurate than those of the onsite pathologist using the same slides. Improving steatosis estimation would reduce rejection of healthy livers and prevent unhealthy livers reaching patients.

Suitable organs require maintenance before transplantation. Organs donated after circulatory death are affected by ischemia, which causes a build-up of succinate. Succinate, in turn, releases reduced flavin mononucleotide (FMH_2_) from mitochondria. Upon reoxygenation during or after transplantation, succinate is rapidly converted to fumarate, and FMNH_2_ to FMN, producing reactive oxygen species that damage cells. Storing organs at low temperatures, known as static cold storage (SCS), slows the build-up of succinate. An alternative to SCS is machine perfusion. The organ is suspended in a sealed bath and supplied nutrients and oxygen via pumps. A 2018 article published in *Nature* by David Nasralla (University of Oxford, Oxford, UK) and colleagues found that normothermic (i.e., 37°C) machine perfusion, compared with SCS, resulted in a 50% reduction in injury to livers and fewer organs discarded before surgery. Building on this work in their *EBioMedicine* Article (October, 2020), Andrea Schlegel (University Hospital of Zürich, Zürich, Switzerland) and colleagues investigated a hypothermic (i.e., 10°C) perfusion in a rat model. Rat livers undergoing hypothermic perfusion released FMN at a lower rate than normothermic perfusion. These so-called cold livers were also healthier after transplantation, showing higher concentrations of ATP (indicating less mitochondrial damage) and less cellular necrosis. The group also found that, for human livers, failed transplants displayed higher concentrations of FMN than successful transplants. This work supports the further development of hypothermic perfusion and the use of FMN as a biomarker for organ quality.

The healthiest of organs can still conceal dangers, for instance B cells from the donor's immune system. B cells can harbour Epstein-Barr virus, the causative agent of cancers such Burkitt lymphoma. Terrance Ku and colleagues (University Health Network, ON, Canada), in our October issue, showed how a machine perfusion set-up can be used to remove these harmful cells from donor lungs. In the study, donated non-transplantable lungs were perfused with rituximab, an αCDC20 antibody, that targets B cells for destruction. Treatment with rituximab resulted in a depletion of donor B cells without damage to lung tissue. The lungs were not ultimately transplanted but the work presents a proof of concept for the removal of harmful cells.

Even with all of these precautions, a new organ can cause complications in the patient receiving it. Recipients are given immunosuppressants to prevent their own immune system destroying the transplant. Dampened immune systems cause changes to patient microbiomes. Marijn Thijssen (Katholieke Universiteit Leuven, Leuven, Belgium) and colleagues published a study in *EBioMedicine* that measured virome changes during liver transplantation in patients with hepatitis B. After immunosuppression, there was a large increase in the proportion of viruses from the *Anelloviridae* family. These non-pathogenic viruses could serve as warnings of post-transplant damage. An abundance of *Anelloviridae* was associated with an increased likelihood of post-transplant infection and acute kidney injury. The virome, therefore, could serve as an indicator for post-transplant health.

Organ donation is perhaps the most generous act of philanthropy one can undertake. Therefore, research that makes these gifts more likely to save another's life should be applauded and encouraged; research that we aim to closely follow and report in *EBioMedicine*.

*EBioMedicine*

